# *Cryptococcus gattii* Meningitis in a Previously Healthy Young Woman: A Case Report

**DOI:** 10.5811/cpcem.2021.5.52344

**Published:** 2021-08-04

**Authors:** Sarabeth Maciey, Chloe Santa Maria, Sachie Oshima, Jennifer A. Newberry

**Affiliations:** * Stanford School of Medicine, Stanford Health Care, Department of Emergency Medicine, Palo Alto, California; † Stanford School of Medicine, Stanford Comprehensive Otolaryngology Clinic, Department of Otolaryngology, Stanford School of Medicine, Palo Alto, California

**Keywords:** Climate change, meningitis, Cryptococcus gattii, headache, emergency medicine

## Abstract

**Introduction:**

*Cryptococcus gattii (C. gatti)* is a rare cause of meningitis in the United States. Outbreaks in new geographic distributions in the past few decades raise concern that climate change may be contributing to a broader distribution of this pathogen. We review a case of *C. gattii* in a 23-year-old woman in Northern California who was diagnosed via lumbar puncture after six weeks of headache, blurred vision, and tinnitus.

**Case Report:**

A 23-year-old previously healthy young woman presented to the emergency department (ED) after multiple visits to primary care, other EDs, and neurologists, for several weeks of headache, nausea, tinnitus, and blurred vision. On examination the patient was found to have a cranial nerve VI palsy (impaired abduction of the left eye) and bilateral papilledema on exam. Lumbar puncture had a significantly elevated opening pressure. Cerebrospinal fluid studies were positive for *C. gattii*. The patient was treated with serial lumbar punctures, followed by lumbar drain, as well as amphotericin and flucytosine. The patient had improvement in headache and neurologic symptoms and was discharged to another facility that specializes in management of this disease to undergo further treatment with immunomodulators and steroids.

**Conclusion:**

Fungal meningitis is uncommon in the US, particularly among immunocompetent patients. Due to climate change, *C. gattii* may be a new pathogen to consider. This finding raises important questions to the medical community about the way global climate change affects day to day medical care now, and how it may change in the future.

## INTRODUCTION

Climate change is expanding the landscape of infectious disease and may impact the daily practice of emergency medicine. *Cryptococcus gattii* (*C gattii*) is an encapsulated yeast whose global distribution has begun changing, possibly due to climate change. This pathogen lives in the soil and in association with certain trees. It was first isolated from eucalyptus but has also been isolated in other tree species in tropical and subtropical geographic distributions, especially Australia and Papua, New Guinea, and to a lesser degree Africa, Europe, Mexico, and South America.^1^ The host is usually infected via the respiratory system from the environment, causing a pneumonia-like illness. This can disseminate to skin and other organs, including the central nervous system. Symptoms of neurologic involvement include headache, fever, neck pain, nausea, vomiting, photophobia, and altered mental status. The incubation period for *C gattii* is not well documented but average symptom onset may be six to seven months from exposure. It is not contagious between people.^1^

In the early 2000s, an outbreak was documented in the Pacific Northwest in primarily human immune-deficiency virus (HIV)-negative patients.^2^ From 1999 to 2007, British Columbia saw an increase in incidence of cases from five per year to 38 per year.^3^ Then from 2010–2012, cases were documented in states outside the Pacific Northwest.^4^ Cases were reported in the following states: Alabama (1 case); California (13); Florida (1); Georgia (5); Hawaii (1); Michigan (1); Montana (1); and New Mexico (2). These cases raised concern that *C gattii* has either adapted to a new climate niche or that climate change has opened new areas that *C gattii* can inhabit.^5^,^6^

## CASE REPORT

A 23-year-old previously healthy woman presented to the emergency department (ED) with nausea and vomiting, weight loss, headache, tinnitus, diplopia, photophobia, dizziness, and paresthesia. Six weeks prior to presentation, the patient started having light-headedness, tinnitus and zigzags in her vision. She then developed nausea, vomiting, and headaches, and over several weeks had multiple visits with her primary care provider, a neurologist, and an outside hospital ED. Outpatient labs and a non-contrast computed tomography (CT) of the head were reportedly negative. She was prescribed a succession of antibiotics for presumed sinusitis, migraine prophylaxis and abortive medications, and received botulinum toxin injections to her forehead, temples, and neck.

On arrival to our ED, the patient reported progressive headache and emotional lability, as well as new numbness and tingling in her left fourth and fifth fingers and right foot. She denied alcohol, smoking, or drug use. Her only reported travel was to Singapore and Taiwan approximately three months prior to symptoms onset. She denied any preceding illnesses. Intake vital signs were as follows: heart rate of 84 beats per minute, blood pressure 107/83 millimeters of mercury, respiratory rate of 20 breaths per minute, temperature of 36.8° Celsius, and pulse oximetry 99% on room air. Physical exam was notable for a supple neck with a full range of motion without pain. Her neurologic exam was notable for a sixth (VI) cranial nerve palsy of the left eye, and subjective decrease in sensation in her left fourth and fifth digits and in her right ankle and foot. While the patient was alert and oriented, her affect fluctuated between animated and flat. On fundoscopic exam, she had pale optic discs bilaterally and papilledema.

**Image 1:**
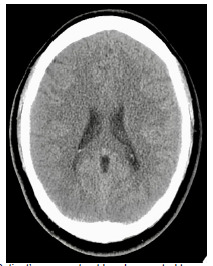
Patient’s non-contrast head computed tomography, with no acute abnormality.

**Image 2:**
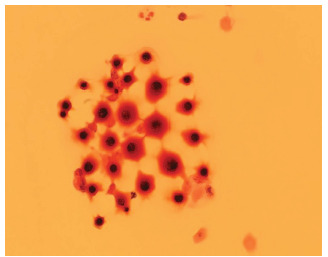
Cerebrospinal fluid Gram stain showing variable-size yeast with narrow-based budding, characteristic of *Cryptococcus gattii*.

Blood tests, including a complete blood count and comprehensive metabolic panel, were notable only for a mildly elevated ammonia (39 micromoles per liter [umol/L] [reference range 11–32 umol/L]). A non-contrast CT of the head was obtained, which was negative for any acute intracranial process (Image 1). A lumbar puncture demonstrated elevated opening pressures of greater than 55 centimeters of water (cm H_2_0) (normal range 5–25 cm H_2_O). Cerebrospinal fluid studies showed 70 white blood cells (normal range 0–5 microliters [uL]); 75% lymphocytes (40–80%); protein 62 milligrams per deciliter (mg/dL) (15–45 mg/dL); glucose 34 mg/dL (40–70 mg/dL), and Gram stain with evidence of fungal elements consistent with *C gattii* (Image 2). Cultures later also grew *C gattii*. The patient was admitted to the neurology service.

**CPC-EM Capsule**

What do we already know about this clinical entity?

Cryptococcus gattii *is a leading cause of meningitis in immunocompetent hosts in tropical/subtropical regions and has a high mortality rate*.

What makes this presentation of disease reportable?

*The rarity and nonspecific presentation of this pathogen require a high level of suspicion by the emergency clinician to reach the diagnosis*.

What is the major learning point?

C. gattii *is a rare pathogen in the United States, but recent outbreaks suggest climate change may contribute to its broader distribution*.

How might this improve emergency medicine practice?

*It is crucial that emergency clinicians consider the effect of the planet’s changing climate on the distribution of disease*.

**Image 3:**
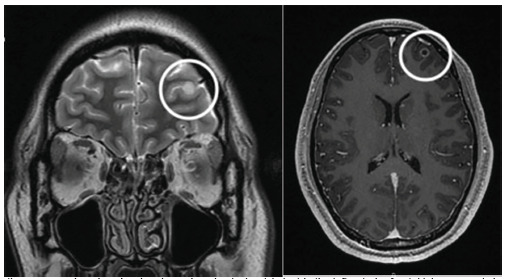
Magnetic resonance imaging showing ring enhancing lesion (circles) in the left anterior frontal lobe; coronal view at left and axial at right.

Magnetic resonance imaging (MRI) showed a 5.6 mm ring-enhancing lesion in the left anterior frontal lobe consistent with a cryptococcoma (Image 3). Additional tiny punctate enhancing lesions in the left caudate head and basal ganglia were also felt to represent cryptococcal disease.

The patient’s symptoms of headache, diplopia, nausea, vomiting and tinnitus gradually improved after induction therapy with amphotericin and flucytosine and multiple lumbar punctures per week, followed by a lumbar drain. After a one-month admission she was discharged to another facility that specializes in immune modulation and steroids. After treatment at the specialty facility, her headache, nausea, and vomiting resolved. Her exam upon discharge was significant only for mild bilateral cranial nerve VI palsies, and her cerebrospinal fluid cultures remained negative for over two weeks. The patient was ultimately discharged from the specialty facility on indefinite fluconazole prophylaxis.

## DISCUSSION

*Cryptococcus gattii* is an encapsulated yeast that has traditionally been found in tropical and subtropical geographic distributions. In more recent years it has also been cultured from eucalyptus trees exported from Australia to California and the Pacific Northwest.^7^ Fungal spores released from the soil and into the air can come in contact with human nasal passageways as a route of inoculation. Globally, it is a leading cause of meningitis in immunocompetent hosts and maintains a high mortality rate.^8^ One study cited 20% of patients with *C gattii* infection dying directly because of the infection, and 13% dying from complicating factors.^9^

Symptoms may occur months to years before clinical diagnosis, as the onset can be quite insidious. Patients may present with a nonspecific assortment of constitutional, respiratory, gastrointestinal, and central nervous system symptoms over several weeks or months. In addition, a non-contrast CT can be negative in as many as 50% of cases, requiring the clinician to maintain a high level of suspicion to continue pursuing a central nervous system etiology.^10^ Computed tomography can show hydrocephalus, hypodense nodules, or diffuse atrophy, although these findings are non-specific.^11^ Our patient’s course fits with this presentation, as she had six weeks of headache with a negative CT as seen above.

This patient had traveled to Singapore and Taiwan three months prior to the onset of symptoms, and both countries have rare, reported cases of *C gattii*. One study in Singapore identified 62 patients with *Cryptococcus* over eight years at a 1400-bed hospital, and only three of those (4.8%) were infected with *C gattii*, the majority being *Cryptococcus neoformans* (*C neoformans*).^12^ Similar prevalence rates have been seen in Taiwan, with 95.9% of Cryptococcus being caused by *C neoformans* and 4.1% by *C gattii*. This is compared to Australia and New Zealand, where 85% of cryptococcal infection are caused by C neoformans and 15% by C gattii.^13^ These data raise the question of whether this patient was exposed to *C gattii* during her travels to one of these countries or at home in northern California. This is especially concerning given that since the late 1990s there has been an increase in the incidence of this pathogen in the Pacific Northwest and subsequently in other states as well, including California.

Cases of *C gattii* infection in the northwestern United States have occurred in Washington and Oregon, and it has been identified in environmental samples in San Francisco, California.^13^ The climates in these areas are characterized by warm, dry summers and mild, wet winters. This climate is different from the traditional tropical climates in which *C gattii* has previously been found. Some hypotheses propose that *C gattii* has existed in the environment of the Pacific Northwest for the past 35 years but that recent climate changes have allowed for higher concentrations to flourish.^14^ Another theory is that this pathogen has adapted to new environmental niches.

## CONCLUSION

*Cryptococcus gattii* is a yeast previously found only in specific geographic and climate distributions. Cryptococcal infections are thought to be the most common cause of fungal meningitis in immunocompetent hosts, although most research has focused on HIV-positive patients.^15^ The onset of symptoms is insidious, the symptoms themselves are often vague and nonspecific, and they may involve multiple systems such as constitutional, respiratory, gastrointestinal, and the central nervous system. This nonspecific presentation, in addition to the pathogen’s rarity, requires a high level of suspicion by the emergency clinician to determine the diagnosis (usually by MRI or lumbar puncture).

Although still rare, this pathogen is becoming more common in new parts of the world such as the Pacific Northwest and California, which may be related to effects of climate change on broadening *C gattii’s* potential habitats. It is becoming increasingly crucial that emergency clinicians consider the implications of the planet’s changing climate on the distribution and manifestation of disease, and as a result consider additional neurologic and infectious workup in young, healthy, and immunocompetent persons with intractable headache and no other objective signs of infection.
